# Postoperative obstruction of recurrent esophageal hiatal hernia: A case report

**DOI:** 10.1097/MD.0000000000041955

**Published:** 2025-04-11

**Authors:** Chao Su, Weifeng Liu, Dandan Lian, Cunchuan Wang

**Affiliations:** a Department of Gastrointestinal Surgery, Weihai Municipal Hospital, Cheeloo College of Medicine, Shandong University, Weihai, China; b Department of Metabolic and Bariatric Surgery, The First Affiliated Hospital of Jinan University, Guangzhou, China; c Department of Pediatric Surgery, Weihai Municipal Hospital, Cheeloo College of Medicine, Shandong University, Weihai, China.

**Keywords:** case report, hiatal hernia, laparoscopic surgery, obstruction, recurrence

## Abstract

**Rationale:**

Surgical repair is considered the optimal treatment for hiatal hernia (HH); however, postoperative complications, such as obstruction, can occur, which may complicate recovery. This case report details a patient who experienced postoperative obstruction following laparoscopic adhesiolysis combined with transabdominal HH repair and Nissen fundoplication for recurrent esophageal HH.

**Patient concerns:**

A 64-year-old female presented with a 3-year history of worsening shortness of breath during physical activity. She also reported upper abdominal pain, postprandial vomiting, and difficulty swallowing, all of which significantly compromised her quality of life. The patient had a 5-year history of diabetes, with no notable family or genetic history. Three years prior, she underwent laparoscopic HH repair at a local hospital, but specific details of that surgery were not available.

**Diagnoses:**

HH was confirmed through computed tomography scans of the chest and upper abdomen, as well as serial examinations of the upper digestive tract.

**Intervention:**

On March 9, 2023, the patient underwent laparoscopic abdominal adhesion release, transabdominal HH repair, and Nissen fundoplication. Postoperatively, she received parenteral nutrition, acid inhibition, and symptomatic treatment for deswelling to alleviate abdominal pain and vomiting. However, the patient was unable to tolerate oral intake due to obstruction.

**Outcomes:**

A contrast study revealed high obstruction at the distal esophagus, specifically at the junction of the stomach and esophagus, likely at the cardia. Subsequently, on April 6, 2023, the patient underwent a second laparoscopic exploration and adhesiolysis under general anesthesia. Postoperatively, the patient recovered well and was discharged on April 14, 2023. During the 12-month follow-up on April 30, 2024, she reported returning to normal daily activities with no complaints of discomfort.

**Lessons:**

This case highlights that laparoscopic adhesiolysis combined with transabdominal HH repair and Nissen fundoplication can effectively address recurrent esophageal HH along with postoperative obstruction. The findings provide important insights for the individualization of surgical procedures for patients with HH.

## 1. Introduction

Hiatal hernia (HH) is a condition where abdominal organs pass through the diaphragmatic esophageal hiatus into the chest cavity and is the most common type of diaphragmatic hernia, accounting for over 90% of cases.^[1]^ Specifically, The gastroesophageal junction and fundus may enter the chest cavity through the hiatus in most patients with HH, which gives rise to a range of digestive difficulties and discomforts.^[1,2]^ Patients with HHs often experience symptoms like acid reflux, burning behind the sternum, and difficulty swallowing, significantly reducing their quality of life.^[2]^ Although benign in nature, HH can inflict considerable pain and impose a substantial burden on both patients and society.^[3]^

Surgery is usually an effective treatment for severe hernias, but recurrence after surgery is sometimes inevitable due to persistent issues with the esophageal hiatus repair.^[1,2]^ When recurrent HHs occur, some cases may progress to postoperative obstruction.^[4]^ Obstruction means that a portion of the esophagus or stomach is blocked or impeded, preventing food and liquids from passing normally.^[5]^ This can lead to severe pain, persistent vomiting, swallowing difficulties, and even malnutrition. The treatment of recurrent HH with postoperative obstruction usually requires a comprehensive evaluation of the patient’s condition, which may include medication, repeat surgical repair, or other treatment options.^[6]^ Therefore, timely diagnosis and professional medical advice are crucial for managing patients with HH, alleviating symptoms, and improving their quality of life.

This case report presented a 64-year-old female with HH, who was treated with laparoscopic adhesiolysis combined with transabdominal hiatal hernia repair and Nissen fundoplication and had successful recovery without recurrence or comiditities.

## 2. Case presentation

### 2.1. Ethical approval

Ethical committee approval was obtained for the review and publication of this case report from the ethical committee of the First Affiliated Hospital of Jinan University, China (No. KY-2024-120). Written informed consent from the patient was obtained to use the photo and case information for professional purposes.

### 2.2. Patient information

The patient was a 64-year-old female with a height of 150 cm, weight of 45 kg, and a body mass index of 20 kg/m². She was admitted to the Neurology Department of the First Affiliated Hospital of Jinan University on February 23, 2023, due to a 10-year history of shortness of breath after activity, which had worsened over the past 3 years. She reported of no symptoms of upper abdominal pain and heartburn. But she complaint of postprandial vomiting, and difficulty swallowing for sometimes.

The patient has a 5-year history of type 2 diabetes, managed with 1 tablet of gliclazide per day, maintaining fasting blood glucose levels between 6.7 and 10.4 mmol/L. Three years ago, she underwent laparoscopic hiatal hernia repair surgery at a local hospital, though the specifics of the procedure are unclear.

### 2.3. Clinical findings and diagnosis

Upon admission, a thorough examination revealed no positive neurological findings. Physical examinations found tenderness in the right epigastrium and rebound tenderness, the rest of the abdomen was normal, vital signs. The patient had a normal temperature of 36.9 °C, along with normal respiration and blood pressure. Cardiac ultrasound and ECG results were normal. Blood glucose monitoring showed levels ranging from 5.4 to 12.6 mmol/L, which did not meet surgical safety standards. Laboratory results were shown in Table 1. On February 25, 2023, a chest and upper abdominal computed tomography scan revealed a large HH (type III) with the herniation of the gastric fundus and part of the greater omentum into the thoracic cavity. The esophageal hiatus appeared enlarged with a diameter of about 5 cm, and there were adhesions around the hernia sac. Both the gastric fundus and the gastroesophageal junction moved upward to the thoracic cavity with mixed density, about 4 cm from the esophageal hiatus.

**Table 1:** Laboratory results of the patient at admission.

Indicators	Laboratory results	Unit	Reference values
White blood cell count	6.73	×10^9^/L	3.5–9.5
Neutrophil percentage	66.30	%	40–75
LYMPH percentage	25.70	%	20–50
Monocyte percentage	5.50	%	3–10
Gel acid granulocyte percentage	2.40	%	0.4–8
Basophil percentage	0.10	%	0–1
Neutrophil absolute value	4.46	×10^9^/L	1.8–6.3
Lymphatic value	1.73	×10^9^/L	1.1–3.2
Monocyte absolute value	0.37	×10^9^/L	0.1–0.6
Gel acidic granulocyte absolute value	0.16	×10^9^/L	0.02–0.52
Basophils absolute value	0.01	×10^9^/L	0–0.06
Red Blood cell count	3.65	×10^9^/L	3.8–5.1
Hemoglobin content	112.00	g/L	115–150
Hematocrit	34.30	%	35–45
Mean red blood cell volume	94.00	fL	82–100
Mean erythrocyte hemoglobin	30.70	pg	27–34
Mean erythrocyte hemoglobin concentration	327.00	g/L	316–354
Red blood cell distribution width (SD)	43.90	fL	–
Red blood cell distribution width (CV)	12.80	%	11.5–15.0
Platelet count	232.00	×10^9^/L	125–350
Mean platelet volume	9.20	fL	8–12.5
Platelet volume	0.21	%	0.16–0.40
Total bilirubin	8.3	µmol/L	5.1–23
Bound bilirubin	1.2	µmol/L	0.6–68
Unbound bilirubin	7.10	µmol/L	1.7–17
Total bile acid	11.4	µmol/L	0–10
Total cholesterol	5.69	µmol/L	3.1–5.7
Triglyceride	1.16	µmol/L	0.56–1.7
High density lipoprotein C	1.20	µmol/L	0.91–2.05
Low density lipoprotein C	3.35	µmol/L	1.57–3.76
Apolipoprotein A	1.46	g/L	1–1.6
Apolipoprotein B	1.15	g/L	0.6–1.08
Lipoprotein a	858.7	mg/L	0–300
Lactate dehydrogenase	227	U/L	109–245
Creatine kinase	60	U/L	26–174
Creatine kinase isoenzyme	15	U/L	3–25
Alpha-hydroxybutyrate dehydrogenase	180	U/L	72–182
Retinol-binding protein	48.3	mg/L	25–70
Glycated serum protein	159.8	umol/L	122–236
Hypersensitive C-reactive protein	1.6	mg/L	Low risk: <1 Moderate risk: 1 High risk: >3 Inflammation: >10
Osmotic pressure	298	mosm	280
Anion gap	7.60	–	–
Glutathione reductive ester	59	U/L	34–73
Alkaline phosphatase	72	U/L	50–135
Cholinesterase	8709	U/L	4000–12,900
Total protein	62.7	g/L	65–85
Albumin	35.1	g/L	35–52
Prealbumin	221.4	mg/L	200–400
Globulin	27.60	g/L	20–40
White Sphere ratio	1.27	–	1.2–2.4
Total bilirubin	8.3	µmol/L	5.1–23
Bound bilirubin	1.20	µmol/L	0.6–6.8
Unbound bilirubin	7.10	µmol/L	1.7–17
Total bile acid	11.40	µmol/L	0–10
Total cholesterol	5.69	mmol/L	3.1–5.7
Triglyceride	1.16	mmol/L	0.56–17
High density lipoprotein C	1.2	mmol/L	0.91–2.05
Low density lipoprotein C	3.35	mmol/L	1.57–3.76
Apolipoprotein A	1.46	g/L	1–1.6
Apolipoprotein B	1.15	g/L	0.6–1.08
Lipoprotein a	858.7	mg/L	0–300
Lactate dehydrogenase	227	U/L	109–245
Creatine creatinase	60	U/L	26–174
Potassium	3.80	mmol/L	3.5–5.3
Sodium	143.2	mmol/L	137–147
Chlorine	105.9	mmol/L	99–110
Total carbon dioxide	29.7	mmol/L	21–31
Glucose	4.77	mmol/L	3.89–6.11
UREA	7.01	mmol/L	1.5–7.5
Creatinine	49.3	µmol/L	32–106
Glomerular filtration rate eGFR	99	ml/min/1.73 m^2^	70–100
URIC acid	342.2	µmol/L	155–360
Cystatin C	0.97	mg/L	0.54–1.15
Calcium	2.14	mmol/L	2–2.8
Inorganic phosphorus	1.32	mmol/L	0.96–1.62
Magnesium	0.88	mmol/L	0.7–1.1
Iron	9.3	µmol/L	9.5–25
Egg day (TRF)	2.400	g/L	2–4
Amylase	83.9	U/L	15–125
Alanine aminotransferase	14	U/L	7–40
Aspartate aminotransferase	16	U/L	13–35
Y-glutamyltransferase	16	U/L	7–45

The patient’s blood glucose levels was controlled between 6.2 and 9.7 mmol/L by taking active glucose lowering therapy during the time period from February 26, 2023 to March 5, 2023. After the glucose levels showed stable status, she was transferred to the Gastrointestinal Surgery Department on March 6, 2023. On March 7, 2023, an upper gastrointestinal series was performed, revealing a type III HH. The contrast study showed the gastric fundus and a portion of the greater omentum protruding through the esophageal hiatus into the thoracic cavity. The esophagogastric junction was displaced above the diaphragm, confirming the hernia’s type and extent.

### 2.4. Surgical interventions

Routine assessments of cardiac and pulmonary function was conducted prior to surgery. Comprehensive preoperative tests that included blood count, electrolyte levels, and liver and kidney function tests were proceeded to evaluate the patient’s overall physical condition. The surgical process and potential risks and complications had been explained to the patient to alleviate any anxiety. The patient was informed of the standard fasting guidelines and followed the following steps: fasting from food after 22:00 the night before surgery and refraining from drinking after 24:00. These procedures were carefully carried out to ensure the surgical indications of the patient and the best physical status for operation.

**Figure 1.**
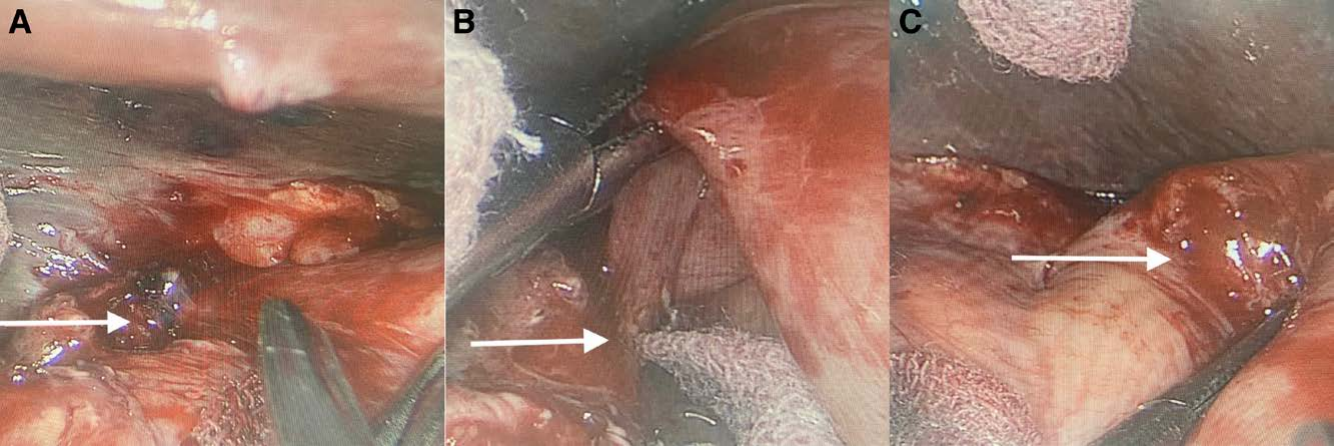
Surgical procedure of surgery for recurrent hiatal hernia. The lessions were pointed out with white arrows. (A) Puncture site. (B) Exposure and suturing of the hiatal hernia. (C) Nissen fundoplication procedure.

Following preoperative preparations, an indwelling gastric tube was placed 30 minutes before surgery. Then, she underwent laparoscopic adhesiolysis, transabdominal hiatal hernia repair, and Nissen fundoplication under general anesthesia on March 9, 2023 (Fig. 1). The first step was to identify and mark the laparoscopic puncture sites (Fig. 1A). Typically, several key positions on the abdomen are selected for puncture to ensure that surgical instruments can enter the abdominal cavity conveniently and provide a good view. Once the laparoscope enters the abdominal cavity, the hernia site was gradually revealed (Fig. 1B) by carefully separating the surrounding tissues. Intraoperative findings included an enlarged esophageal hiatus with a hernia ring approximately 6 × 6 cm, with the gastric fundus and part of the greater omentum entering the hernia sac. Adhesions around the hernia sac were meticulously dissected using an ultrasonic scalpel, the previous mesh was cut, the hernia sac was fully dissected, the diaphragmatic crura were sutured with silk, the esophageal hiatus was reduced in size, and the gastric fundus was folded and sutured using absorbable sutures. The Nissen fundoplication procedure requires careful handling to ensure that the wrap is of appropriate tightness, avoiding difficulties in swallowing and alleviating symptoms such as heartburn. During the surgery, the top of the stomach (fundus) was wrapped around the lower end of the esophagus and sutured in place, forming a 360° wrap (Fig. 1C). To prevent recurrence of the hernia, the edges of the hiatus was sutured, and a patch was used to reinforce the area, ensuring that the hiatus was securely closed.

Postoperatively, the patient experienced persistent paroxysmal upper abdominal pain and vomiting after meals, preventing oral intake. She was managed with parenteral nutrition, acid suppression, and anti-inflammatory symptomatic treatment. Additionally, the patient was advised on postoperative care instructions. Esomeprazole 40 mg bid was administered postoperatively until March 22. The patient began parenteral nutrition on March 9, 2023: long chain fat milk 250 mL + compound amino acid (18aa) 200 mL + 10% glucose 500 mL + alanyl glutamine 50 mL + potassium aspartate 20 mL + multivitamin 5 mL qd were intravenously taken until March 28, 2023. The gastric tube was still retained until March 13 after surgery, because the patient had no obstruction after eating a slag-free liquid diet and had obstruction after eating a slag diet. The enteral nutrition (energy) was given orally 1000 mL until March 20, 2023.

### 2.5. Postoperative obstruction and further interventions

**Figure 2.**
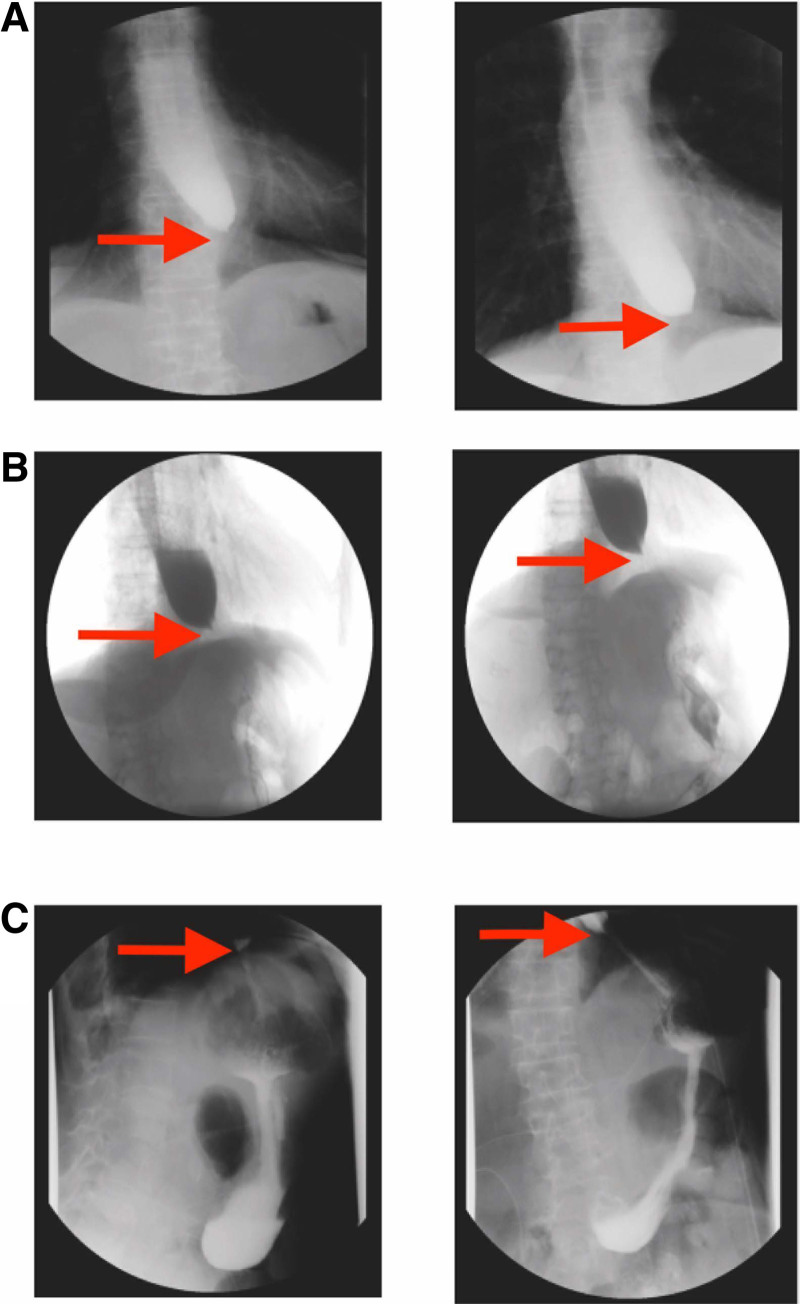
Gastrointestinal contrast study. The lessions were pointed out with white arrows. (A) The contrast study on March 27, 2023 revealed significant narrowing of the distal esophagus, with delayed passage of the contrast medium. (B) The upper gastrointestinal contrast study on 3 April indicated narrowing at the lower end of the esophagus, with difficulty in passing the contrast agent. (C) The upper gastrointestinal contrast study on April 10, 2023 showed smooth passage of the contrast agent, with no evidence of narrowing.

From March 13 to March 26, the patient was unable to eat food with residue, and ate a small amount of liquid without residue. The patient was unable to have a normal diet. On March 27, 2023 and April 3, 2023, a follow-up upper gastrointestinal series indicated an esophageal stricture, with difficulty passing the contrast agent (Fig. 2A and B). The contrast study showed significant narrowing at the distal esophagus, with delayed passage of the contrast medium, suggesting a stricture at the junction of the stomach and esophagus (possibly at the site of cardia). On April 5, 2023, an attempted balloon dilation under gastroscopy was unsuccessful. The endoscopic view showed a tight stricture at the distal esophagus, preventing the passage of the balloon catheter. On April 6, 2023, she underwent a second laparoscopic exploration and adhesiolysis under general anesthesia. Famotidine 20 mg bid was administered postoperatively until 11 April. Parenteral nutrition started from April 7th, 2023. Glucose sodium chloride injection 500 mL + 5% glucose injection 500 mL + sodium potassium magnesium calcium injection 500 mL + compound amino acid (18aa) 200 mL + potassium aspartate 20 mL + alanyl glutamine 50 mL + multivitamin 5 mL qd were intravenously taken until April 11th, 2023. For the second surgery treatment for the postoperative obstruction, gastric tube indwelling was placed from March 27, 2023 to April 11, 2023. Postoperatively, her upper abdominal pain improved, and she experienced no obstruction sensation when consuming a residue-free liquid diet. On April 10, 2023, a follow-up upper gastrointestinal series showed smooth passage of the contrast agent through the distal esophagus, without evidence of stricture (Fig. 2C). The contrast study confirmed a patent esophagogastric junction with no significant narrowing or delayed passage.

From April 11, 2023, the patient gradually transitioned to a semiliquid diet without obstruction sensation, had good bowel movements, and normal routine blood tests and liver and kidney function tests. She was discharged on April 14, 2023. Post-discharge, she was prescribed oral esomeprazole 20 mg twice daily for 1 week. She was advised to continue a semiliquid diet for half a month, rest, and avoid strenuous activities. Over a follow-up period of more than 2 months, she reported no abdominal pain, swallowing difficulties, acid reflux, or retrosternal burning sensation, and had returned to normal daily activities. Until the last follow-up by September 2, 2024 vie telephone, she reported that she had a normal diet, without abdominal pain, heartburn or eating difficulties.

## 3. Discussion

Recurrent HH itself is uncommon in the general population, and postoperative obstruction in recurrent HH is a very rare and complex condition. Since the introduction of laparoscopic simple suture repair in 1991, this surgical method has shown good outcomes in repairing recurrent hernias.^[7]^ However, compared to the 15% to 20% recurrence rate associated with open surgery, the recurrence rate for simple suture closure of the hiatal defect has soared to 42%.^[8,9]^ As reported, simple suture repair without a patch is an independent factor influencing postoperative folding, displacement, and hernia recurrence.^[8]^ The lack of strong fascia near the hiatus, followed with sutures primarily surrounding the muscles of the diaphragmatic crura, may cause risk of rupture in the repair, thus, leading to the recurrence during the continuous movement of the diaphragm and the effects of negative pressure in the chest cavity and positive pressure in the abdomen.^[8,9]^ The patient reported in this case underwent the first operation in another hospital without the details of the operation, and the recurrence was diagnosed in our hospital 3 years later. Therefore, it is difficult to confirm the factors related to the recurrence of HH in the current case report.

Patch repair of hiatal hernia can significantly reduce short-term recurrence rates,^[10]^ and guidelines strongly recommend the use of patches to minimize short-term recurrence.^[11,12]^ Approximately 37% of cases indicated that patch repair is suitable for larger defects (>5 cm).^[13]^ Previous researches indicate that surgeons primarily assess the hiatal surface area (HSA) to determine the indications for using a patch.^[14,15]^ HSA was used to classify hiatal hernias based on long-term outcomes, categorizing them as small hernias (HSA < 10 cm²), large hernias (10–20 cm²), and giant hernias (>20 cm²). In hernias with an HSA of 10–20 cm², the recurrence rate sharply increased with primary repair.^[14]^ Therefore, it is recommended that small hernias undergo suture hernia repair only, while giant hernias undergo patch hernia repair.^[14,15]^ This case revealed a large recurrent HH (type III) with an esophageal hiatus enlarged to a diameter of about 5 cm, which is suitable for patch repair. Therefore, transabdominal hiatal hernia repair was proceeded for this patient. Surgeons should balance the benefits and associated risks of patch repair and make the optimal clinical treatment decisions accordingly.^[16,17]^ Currently, there is a lack of unified standards for patch shape.^[16–18]^ Choice of patch position: At the posterior part of the esophageal hiatus, where there are no important anatomical structures, and where there is minimal movement in the entire hiatus area, the patch can be placed with maximum overlap and extension.^[16–18]^

The most common postoperative complaint is difficulty swallowing (84%), followed by weight loss (14%), and others including gastroesophageal reflux symptoms and bleeding.^[16–19]^ Wound infection and pleural fluid collection were the most significant postoperative complications for patients with HH.^[4–6]^ Obstruction after operation in HH patients was very rare. Once obstruction occurs, immediate intervention should be provided.^[4–6]^ Previous studies showed that, bowel obstruction was most prevalent in postoperative period in HH patients.^[4,20]^ Obstruction is a indication for an emergent surgery,^[21]^ that the patient in this report received the second operation immediately after diagnosis of obstruction. Postoperative gastric outlet obstruction was reported only on a 45-year-old female in 2022, which was relieved successfully by gastrojejunostomy.^[22]^ However, no other relevant literatures were found about this situation. Nausea and vomiting were common symptoms of gastric outlet obstruction.^[22]^ The patient in this case report complained of abdominal pain and vomiting after eating after surgery. Furthermore, the patient had no obstruction after eating a slag-free liquid diet and had obstruction after eating a slag diet. Then the patient was found of high obstruction at the junction of the stomach and esophagus (possibly at the site of cardia) and she underwent a second laparoscopic exploration and adhesiolysis under general anesthesia to relieve the obstruction.

Duodenal and type 3 gastric ulcers may be the most common causes of gastric outlet obstruction. But a previous case showed a paraesophageal hiatal herniation of gastric antrum after operation for HH caused gastric outlet obstruction due to the ignoring the physiological characteristics of the patient.^[23]^ The reason for the obstruction in the current case report may be the overfolding of the gastric fundus, which led to esophagogastric junction stenosis. The related reference is rarely reported. Based on our experience on this case, we recommend that surgeons pay some attention to the folding of the gastric fundus during the operation.

## 4. Conclusion

In summary, gastric outlet obstruction after the operation for the recurrent HH was rarely reported. In this case report, laparoscopic adhesiolysis combined with transabdominal hiatal hernia repair and Nissen fundoplication was effective in treating this special case of recurrent esophageal hiatal hernia along with postoperative obstruction. Surgeons should pay some attention to the folding of the gastric fundus during the operation. Surgeons should try every effort to avoid the occurrence of postoperative obstruction to a greater extent based on preoperative assessments and intraoperative conditions. However, this case report only summarize the experience of the patient with special situation. The experience is empirical and can only provide some reference for surgeons. Personalized options should be taken based on different situations of HH patients with postoperative obstructions.

## Author contributions

**Conceptualization:** Chao Su, Cunchuan Wang.

**Data curation:** Chao Su.

**Formal analysis:** Chao Su.

**Investigation:** Weifeng Liu, Dandan Lian.

**Visualization:** Weifeng Liu, Dandan Lian, Cunchuan Wang.

**Writing – original draft:** Chao Su, Weifeng Liu, Dandan Lian.

**Writing – review & editing:** Cunchuan Wang.

## References

[1] Cocco AM Chai V Read M. Percentage of intrathoracic stomach predicts operative and post-operative morbidity, persistent reflux and PPI requirement following laparoscopic hiatus hernia repair and fundoplication. Surg Endosc. 2023;37:1994–2002. 36278994 10.1007/s00464-022-09701-0 PMC10017603

[2] Iwasaki H Tanaka T Miyake S Yoda Y Noshiro H. Postoperative hiatal hernia after minimally invasive esophagectomy for esophageal cancer. J Thorac Dis. 2020;12:4661–9. 33145039 10.21037/jtd-20-1335 PMC7578511

[3] Krawiec K Szczasny M Kadej A Piasecka M Blaszczak P Głowniak A. Hiatal hernia as a rare cause of cardiac complications—case based review of the literature. Ann Agric Environ Med. 2021;28:20–6. 33775064 10.26444/aaem/133583

[4] Li J Wang Y Shao X. Presentation and management of post-esophagectomy or Gastrectomy Hiatal Hernia. Hernia. 2024;28:1889–97. 39066882 10.1007/s10029-024-03115-8

[5] Tawfik A Thomas AJ Menias CO. Trans-diaphragmatic pathologies: anatomical background and spread of disease on cross-sectional imaging. Curr Probl Diagn Radiol. 2021;50:252–61. 32624297 10.1067/j.cpradiol.2020.05.007

[6] Lalezari S Hanak CR Husted T. Nonoperative management of complicated hiatal hernia after transhiatal esophagectomy- case report. Ann Med Surg (Lond). 2018;33:13–5. 30101007 10.1016/j.amsu.2018.07.007 PMC6083815

[7] Cuschieri A Shimi S Nathanson LK. Laparoscopic reduction, crural repair, and fundoplication of large hiatal hernia. Am J Surg. 1992;163:425–30. 1532701 10.1016/0002-9610(92)90046-t

[8] Zaninotto G Portale G Costantini M. Objective follow-up after laparoscopic repair of large type III hiatal hernia. Assessment of safety and durability. World J Surg. 2007;31:2177–83. 17726627 10.1007/s00268-007-9212-2

[9] Watson DI. Evolution and development of surgery for large paraesophageal hiatus hernia. World J Surg. 2011;35:1436–41. 21380582 10.1007/s00268-011-1029-3

[10] Zhang C Liu D Li F. Systematic review and meta-analysis of laparoscopic mesh versus suture repair of hiatus hernia: objective and subjective outcomes. Surg Endosc. 2017;31:4913–22. 28523363 10.1007/s00464-017-5586-x PMC5715047

[11] Furnee E Hazebroek E. Mesh in laparoscopic large hiatal hernia repair: a systematic review of the literature. Surg Endosc. 2013;27:3998–4008. 23793804 10.1007/s00464-013-3036-y

[12] Kohn GP Price RR Demeester SR. Guidelines for the management of hiatal hernia. Surg Endosc. 2013;27:4409–28. 24018762 10.1007/s00464-013-3173-3

[13] Huddy JR Markar SR Ni MZ. Laparoscopic repair of hiatus hernia: Does mesh type influence outcome? A meta-analysis and European survey study. Surg Endosc. 2016;30:5209–21. 27129568 10.1007/s00464-016-4900-3

[14] Granderath FA Schweiger UM Pointner R. Laparoscopic antireflux surgery: tailoring the hiatal closure to the size of hiatal surface area. Surg Endosc. 2007;21:542–8. 17103275 10.1007/s00464-006-9041-7

[15] Grubnik VV Malynovskyy AV. Laparoscopic repair of hiatal hernias: new classification supported by long-term results. Surg Endosc. 2013;27:4337–46. 23877759 10.1007/s00464-013-3069-2

[16] Aiolfi A Cavalli M Saino G. Laparoscopic posterior cruroplasty: a patient tailored approach. Hernia. 2022;26:619–26. 32335756 10.1007/s10029-020-02188-5

[17] Wang Z Bright T Irvine T Thompson SK Devitt PG Watson DI. Outcome for asymptomatic recurrence following laparoscopic repair of very large hiatus hernia. J Gastrointest Surg. 2015;19:1385–90. 25822063 10.1007/s11605-015-2807-2

[18] Braghetto I Korn O Rojas J Valladares H Figueroa M. Hiatal hernia repair: prevention of mesh erosion and migration into the esophagogastric junction. Arq Bras Cir Dig. 2020;33:e1489. 32428134 10.1590/0102-672020190001e1489 PMC7236328

[19] Antonakis F Kockerling F Kallinowski F. Functional results after repair of large hiatal hernia by use of a biologic mesh. Front Surg. 2016;3:16. 27014698 10.3389/fsurg.2016.00016 PMC4783575

[20] Oppelt PU Askevold I Hörbelt R. Trans-hiatal herniation following esophagectomy or gastrectomy: retrospective single-center experiences with a potential surgical emergency. Hernia. 2022;26:259–78. 33713205 10.1007/s10029-021-02380-1 PMC8881432

[21] Ceccarelli G Pasculli A Bugiantella W. Minimally invasive laparoscopic and robot-assisted emergency treatment of strangulated giant hiatal hernias: report of five cases and literature review. World J Emerg Surg. 2020;15:37. 32487136 10.1186/s13017-020-00316-1 PMC7268602

[22] Li Z Xie F Zhu L Sun L. Post-operative gastric outlet obstruction of giant hiatal hernia repair: a case report. BMC Gastroenterol. 2022;22:47. 35123402 10.1186/s12876-022-02117-z PMC8817466

[23] Coskun S Soylu L Sahin M Demiray T. Gastric outlet obstruction secondary to paraesophageal herniation of gastric antrum after laparoscopic fundoplication. Asian J Surg. 2015;38:117–9. 25813602 10.1016/j.asjsur.2012.11.002

